# Identification of key genes affecting ventilator-induced diaphragmatic dysfunction in diabetic mice

**DOI:** 10.3389/fgene.2024.1387688

**Published:** 2024-05-09

**Authors:** Rongchun Xing, Haibo Yu, Jiangtao Yu, Rong Zeng, Zhijun Xiang, Haoli Ma, Gang Li, Yan Zhao

**Affiliations:** ^1^ Emergency Center, Zhongnan Hospital of Wuhan University, Wuhan, Hubei, China; ^2^ The First College of Clinical Medical Science, Three Gorges University, Yichang, China; ^3^ Department of Biological Repositories, Zhongnan Hospital of Wuhan University, Wuhan, China; ^4^ Yichang Central People’s Hospital, Yichang, Hubei, China; ^5^ Hubei Clinical Research Center for Emergency and Resuscitation, Zhongnan Hospital of Wuhan University, Wuhan, Hubei, China

**Keywords:** mechanical ventilation (MV), ventilator-induced diaphragmatic dysfunction (VIDD), diabetes, RNA-seq, mRNA

## Abstract

**Background:**

Mechanical ventilation (MV) is often required in critically ill patients. However, prolonged mechanical ventilation can lead to Ventilator-induced diaphragmatic dysfunction (VIDD), resulting in difficulty in extubation after tracheal intubation, prolonged ICU stay, and increased mortality. At present, the incidence of diabetes is high in the world, and the prognosis of diabetic patients with mechanical ventilation is generally poor. Therefore, the role of diabetes in the development of VIDD needs to be discovered.

**Methods:**

MV modeling was performed on C57 mice and DB mice, and the control group was set up in each group. After 12 h of mechanical ventilation, the muscle strength of the diaphragm was measured, and the muscle fiber immunofluorescence staining was used to verify the successful establishment of the MV model. RNA sequencing (RNA-seq) method was used to detect mRNA expression levels of the diaphragms of each group, and then differential expressed gene analysis, Heatmap analysis, WGCNA analysis, Venn analysis, GO and KEGG enrichment analysis were performed. qRT-PCR was used to verify the expression of the selected mRNAs.

**Results:**

Our results showed that, compared with C57 control mice, the muscle strength and muscle fiber cross-sectional area of mice after mechanical ventilation decreased, and DB mice showed more obvious in this respect. RNA-seq showed that these differential expressed (DE) mRNAs were mainly related to genes such as extracellular matrix, collagen, elastic fiber and Fbxo32. GO and KEGG enrichment analysis showed that the signaling pathways associated with diabetes were mainly as follows: extracellular matrix (ECM), protein digestion and absorption, PI3K-Akt signaling pathway, calcium signaling pathway, MAPK signaling pathway and AGE-RAGE signaling pathway in diabetic complications, etc. ECM has the closest relationship with VIDD in diabetic mice. The key genes determined by WGCNA and Venn analysis were validated by quantitative real-time polymerase chain reaction (qRT-PCR), which exhibited trends similar to those observed by RNA-seq.

**Conclusion:**

VIDD can be aggravated in diabetic environment. This study provides new evidence for mRNA changes after mechanical ventilation in diabetic mice, suggesting that ECM and collagen may play an important role in the pathophysiological mechanism and progression of VIDD in diabetic mice, and provides some clues for the research, diagnosis, and treatment of VIDD in diabetic context.

## 1 Background

Mechanical ventilation (MV) can maintain alveolar ventilation, increase lung volume, maintain oxygenation, and reduce respiratory work, which is a life-saving measure. However, long-term mechanical ventilation can lead to ventilator-induced diaphragmatic dysfunction (VIDD), which is manifested as diaphragmatic atrophy, decreased contractile force and progressive aggravation, and difficulty in exiting ventilator, which is closely related to poor clinical prognosis (Decramer and Gayan-Ramirez, 2004).

Diabetes is one of the most common metabolic diseases, with a high incidence worldwide, and the prevalence of type 2 diabetes is increasing alarmingly worldwide, with explosive increases in low-income countries and among adolescents/young adults, and with serious implications for longevity and quality of life. The diagnosis, prevention and treatment of the disease face great challenges (Saeedi et al., 2019; Izzo et al., 2021). Clinically, some critically ill patients often need mechanical ventilation. If these critically ill patients have diabetes combined with mechanical ventilation, the prognosis is generally poor, and many studies have shown that diabetes can cause diaphragmatic dysfunction (Van Lunteren and Moyer, 2013). Existing studies on the mechanisms of diabetes-induced muscle damage mostly focus on mitochondrial oxidative stress (Yan et al., 2011; Anderson, 2015). Diabetes may be an important factor in aggravating VIDD. However, previous studies on VIDD models were mostly based on wild-type healthy mouse or rat MV models (Azuelos et al., 2015; Ichinoseki-Sekine et al., 2021; Nakai et al., 2023), and the current omics studies were mostly focused on rat models (Liu et al., 2021a). So far, there has been no study on transcriptomic analysis of diaphragm in MV model of diabetic mice.

In order to understand the changes in mRNA levels caused by mechanical ventilation in patients with diabetes, we used a unique experimental ICU model. Firstly, BKS-DB (DB) mice were selected as the diabetes model (BKS-DB mice were severely obese with significant and continuous glucose increase after Lepr gene was knocked out on C57BL/6JGpt mice. Islet atrophy, which may be accompanied by diabetic nephropathy, diabetic retinopathy, and other symptoms). Studies have shown that diaphragmatic dysfunction can occur in mice after 6–12 h of mechanical ventilation, and there is a significant decrease in diaphragmatic contractility after 6 h of mechanical ventilation. After 12 h of ventilation, the diaphragmatic tissue of mice exhibits significant pathological changes and muscle fiber atrophy. Thus, we chose 12 h as the mechanical ventilation time for mice (Picard et al., 2012a; Mrozek et al., 2012; Matecki et al., 2016).Then, BKS-DB diabetic mice were given mechanical ventilation for 12 h and diaphragm mRNA expression levels were detected by high-throughput sequencing, compared with C57BL/6J (C57) control mice. Finally, we verified the expression of the selected mRNAs using qRT-PCR. We found that mRNA expression changes after MV in diabetic mice, and these changes may shed light on the physiological and pathophysiological processes that occur after MV in diabetic mice, as well as key signaling pathways involved in these genes, which may provide potential therapeutic targets for VIDD.

## 2 Methods

### 2.1 Animals

A total of 44 adult SPF (specific pathogen-free) grade male BKS-DB and C57BL/6J mice were obtained from Gempharmatech (Gempharmatech Co., LTD., Chengdu, China). Animal experiments were conducted in the Biosafety Level III Laboratory of Wuhan University (Wuhan, Hubei, China). The mice were kept in cages with the right temperature (23°C ± 2°C) and humidity (50% ± 5%), light and dark cycles every 12 h. The mice had free access to water and food. All animal experiments were approved by the Ethics Committee of the Animal Laboratory Center of Zhongnan Hospital of Wuhan University and followed the National Institutes of Health guidelines for the care and use of laboratory animals.

Our manuscript confirming the study is reported in accordance with ARRIVE guidelines. All animal experimental procedures were approved by the Institutional Animal Care and Use Committee of Wuhan University (Number: ZN2021189).

### 2.2 Establishing mechanical ventilation model

The MV model was established based on the findings reported in several previous articles (Picard et al., 2012a; Mrozek et al., 2012; Matecki et al., 2016). The mice were anesthetized with sodium pentobarbital (50 mg/kg body weight) via intraperitoneal injection and placed on a constant temperature blanket set at 37°C. Mice in the experimental group (MV group) were orally intubated with a 22-gauge angiocatheter and connected to a small animal ventilator (Vent Elite Harvard Apparatus, United States) where a mixture of air and oxygen was heated, humidified, and delivered into the mice’s respiratory tracts. Volumetric ventilation mode was adopted with a respiratory rate of 150 times/min, a suction/breathing ratio of 1:1, a tidal volume of 8 mL/kg, and mechanical ventilation for 12 h. Mice in the control group, subjected to sham surgery, maintained spontaneous respiration. After 12 h of mechanical ventilation, the mice were euthanized by intraperitoneal injection pentobarbital sodium (150 mg/kg), and the diaphragmatic tissue was taken after the mice were completely unconscious, and 5 diaphragmatic muscle specimens from each group were collected and stored at −80°C for RNA-seq analysis. In addition, six mice were sampled from each group, and a portion of each mouse was used to measure the muscle strength of the diaphragm. The remaining part was stored in muscle fixating fluid and used for fast and slow muscle immunofluorescence analysis.

### 2.3 Measurement of diaphragmatic muscle strength

Take the diaphragm muscle strip (4–6 mm in length and 3–4 mm in width) and quickly immersed it in a constant temperature water bath of Krebs-Henseleit (25°C, pH 7.4) solution. Maintain a continuous supply of a gas mixture consisting of 95% oxygen and 5% carbon dioxide into the water bath. The muscle strip was connected to the muscle force receptor, and the voltage was set to 12 V. Initially, the muscle strip was stimulated with a low frequency (20 HZ), and the baseline was adjusted to find the optimal initial length (cm) of the muscle strip. Subsequently, the diaphragm muscle strips were stimulated with different frequencies (20 HZ, 40 HZ, 80 HZ and 120 HZ), and the corresponding muscle contraction force following stimulation at each frequency was measured. At the end, the weight of the muscle strip is determined in grams (g). Using a muscle density of 1.06 (g/cm³), calculate the cross-sectional area (cm^2^) of each muscle strip as follows: the optimal initial length = wet weight (g)/density (g/cm³)/muscle strip length (cm). Finally, the ultimate muscle force (F) of each mouse’s diaphragm was standardized as: contractile force (g)/cross-sectional area (cm^2^).

### 2.4 Immunofluorescence staining

Immunofluorescence staining of diaphragm tissue was performed to assess the cross-sectional areas (CSAs) of muscle fibers. The diaphragm specimen was extracted from the animal and fixed using an environmentally friendly muscle fixation solution (Servicebio, Wuhan, China). The diaphragm tissue was embedded in paraffin wax and then transverse sliced with a paraffin microtome (RM 2016, Leica Instrument Co., LTD., Shanghai, China). An environmentally friendly dewaxing solution (Servicebio, Wuhan, China) was used for dewaxing. After washing, the antigen was extracted with EDTA repair solution and washed 3 times with PBS. The slices were put into 3% hydrogen peroxide solution, incubated at room temperature for 25 min away from light, blocked endogenous peroxidase, and then washed with PBS for 3 times. Sections were incubated overnight with mouse anti-fast myosin heavy chain antibodies (Servicebio, Wuhan, China) and anti-slow myosin heavy chain antibodies (Servicebio, Wuhan, China) at 4°C. Then the corresponding second antibody were applied, and the sections were incubated at room temperature for 50 min, followed by a TBST wash. After slightly dried, the slices were used for DAPI staining analysis, and finally the slices were sealed with anti-fluorescence quenching agent (Servicebio, Wuhan, China). Images were captured using the microscope (OLYMPUS IX73, Olympus Co., Japan) and cellsens standard software. The determination of CSAs was performed using Image J software (V1.52a, National Institutes of Health, United States).

### 2.5 Analysis of RNA sequencing data

In each of the DB-CON, DB-MV, C57-CON, and C57-MV groups, five samples of diaphragmatic tissue were collected. Total RNA was extracted from diaphragm tissue with TRIzol reagent (Invitrogen, Carlsbad, CA, United States) as recommended in the instructions and genomic DNA was removed using DNase I (TaKara, Beijing, China). The purity and integrity of the RNA were assessed using the NanoDrop (Thermo Fisher) and Bioanalyzer 2100 systems (Agilent Technologies, United States). RNA-seq transcriptome library was prepared following TruSeq RNA sample preparation Kit from Illumina (San Diego, CA) with 1 μg of total RNA for each. The raw paired end reads were trimmed and quality controlled by SeqPrep and Sickle with default parameters. Reference genome and gene model annotation files were downloaded directly from the genome website. Alignment to the Reference genome was performed using Hisat2 (v2.0.1). The mapped reads of each sample were assembled and quantified gene abundances by StringTie. To identify DEGs (differential expressed genes) between two groups, the expression level of each transcript was calculated according to the transcripts per million reads (TPM) method. The differentially expressed mRNA was identified with R-package DESeq2 by |log2 (fold change)| > 0.58 and *p*-value < 0.05. Then differential gene statistical analysis, heatmap analysis, WGCNA analysis, Venn analysis, GO and KEGG enrichment analysis were performed.

### 2.6 Quantitative real-time polymerase chain reaction verification in VIDD mice

The expression levels of 15 most critical genes (Eln, Mfap5, Pcolce, Fbn1, Fstl1, CD248, Col1a1, Col1a2, Col3a1, Col5a1, Col6a1, Col6a2, Col6a3, Col15a1, Fbxo32) selected by the analysis of RNA-seq were validated by qRT-PCR using three samples in each of the four groups. Total RNA was extracted from diaphragm tissue using a mirVana miRNA Isolation Kit (Ambion, Austin, TX, United States) according to the manufacturer’s protocol, and the concentration and purity were assayed using a NanoDrop spectrometer (Thermo Fisher Scientific, Inc.). A total of 1 µg of total RNA was used to synthesize cDNA using a ReverTra Ace qPCR RT Kit (cat. no. FSQ-101; Toyobo Life Science, Osaka, Japan; Code No. FSQ-101). The RNA samples were mixed with RT kit reagents and incubated at 37°C for 15 min and 99°C for 5 min. Finally, the samples were diluted in diethylpyrocarbonate-treated water and stored at-20°C. cDNA was amplified and recorded using SYBR Green Real-Time PCR Master Mix (cat. no. QPK-201; Toyobo Life Science) on a CFX Connect Real-Time PCR System (Bio-Rad; CFX Maestro 1.0 software). All primers were synthesized by Genscript Biotech Corporation, Inc., and the sequences are shown in Supplementary Table S1. The expression of the selected mRNAs was normalized to that of the housekeeping gene GAPDH. The thermocycling program for PCR consisted of 40 cycles of 5 s at 95°C, 10 s at 55°C, and 15 s at 72°C, followed by a final extension at 72°C for 5 min and a melt curve analysis from 65°C to 95°C. The data were analyzed using the 2^−ΔΔCT^ method (Schmittgen and Livak, 2008).

### 2.7 Statistical analysis

In addition to transcriptome data, the data of diaphragm muscle strength and muscle fiber cross-sectional area were showed as mean ± standard deviation (SD). Student’s t tests were used for comparisons between two groups. The data were analyzed using Prism software (v8.0.1, SanDiego, United States), and *p*-value < 0.05 was considered significant.

## 3 Results

### 3.1 Diaphragmatic muscle strength measurement

**FIGURE 1:**
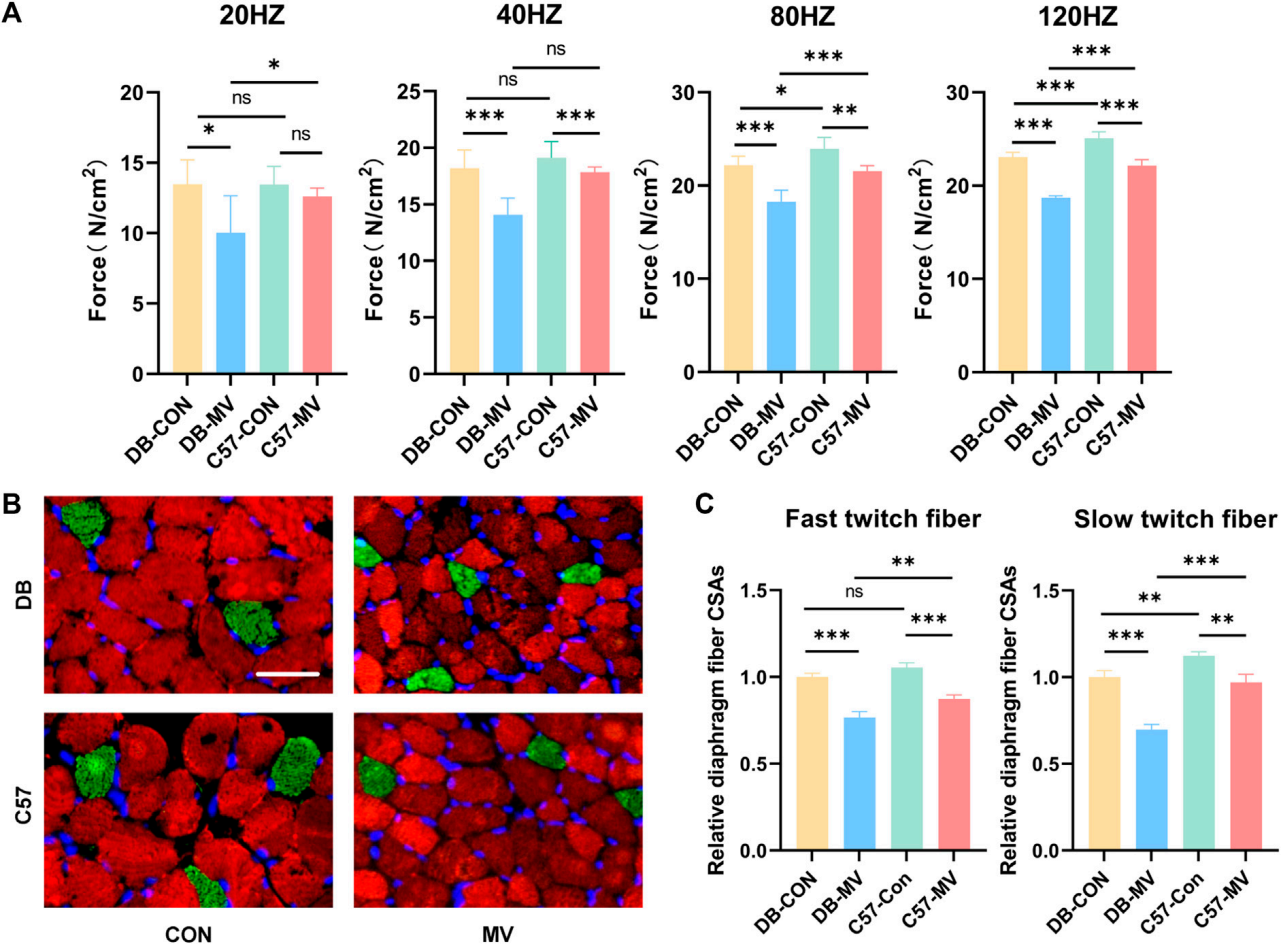
The changes of diaphragmatic muscle strength, immunofluorescence staining images and relative diaphragmatic cross-sectional area of mice in each group after mechanical ventilation were stimulated at different frequencies. **(A)** Changes of muscle strength of diaphragm under different frequency stimulation (*n* = 6 in each group); **(B)** Representative immunofluorescence staining of fast-twitch fibers (red) and slow-twitch fibers (green) in diaphragmatic cross-sectional area in each group (*n* = 3 in each group), scale bar, 50 μm; **(C)** Comparison of changes in relative diaphragmatic cross-sectional area after mechanical ventilation in DB and C57 mice. The relative cross-sectional area of each group was normalized by the DB-CON group. Scale bar = 50 μm. Groups: DB-CON, DB-control group; DB-MV, DB-mechanical ventilation group; C57-CON, C57-control group; C57-MV, C57-mechanical ventilation group. “*, ** and ***” are separately indicates *p*-values < “0.05, 0.01 and 0.001.” The error bars indicate the ±SD.

The diaphragmatic muscle strength of DB and C57 mice in MV group and control group were compared under different frequency stimulation (20 HZ, 40 HZ, 80 HZ and 120 HZ). The diaphragmatic muscle strength of DB and C57 mice decreased after mechanical ventilation compared with their respective controls (Figure 1A). Compared with C57 mice, DB mice experienced a more significant decrease in diaphragmatic muscle strength after mechanical ventilation (Figure 1A).

### 3.2 Diaphragm immunofluorescence results

The cross-sectional area of diaphragmatic fibers in DB and C57 mice was compared between MV group and control group. The transverse area of the diaphragm fibers in both DB and C57 mice decreased after mechanical ventilation compared to the control group (Figures 1B, C). Compared with C57 mice, the transverse area of the diaphragm fibers of DB mice was more significantly reduced after mechanical ventilation (Figures 1B, C).

### 3.3 RNA-seq in diaphragm tissue

**FIGURE 2:**
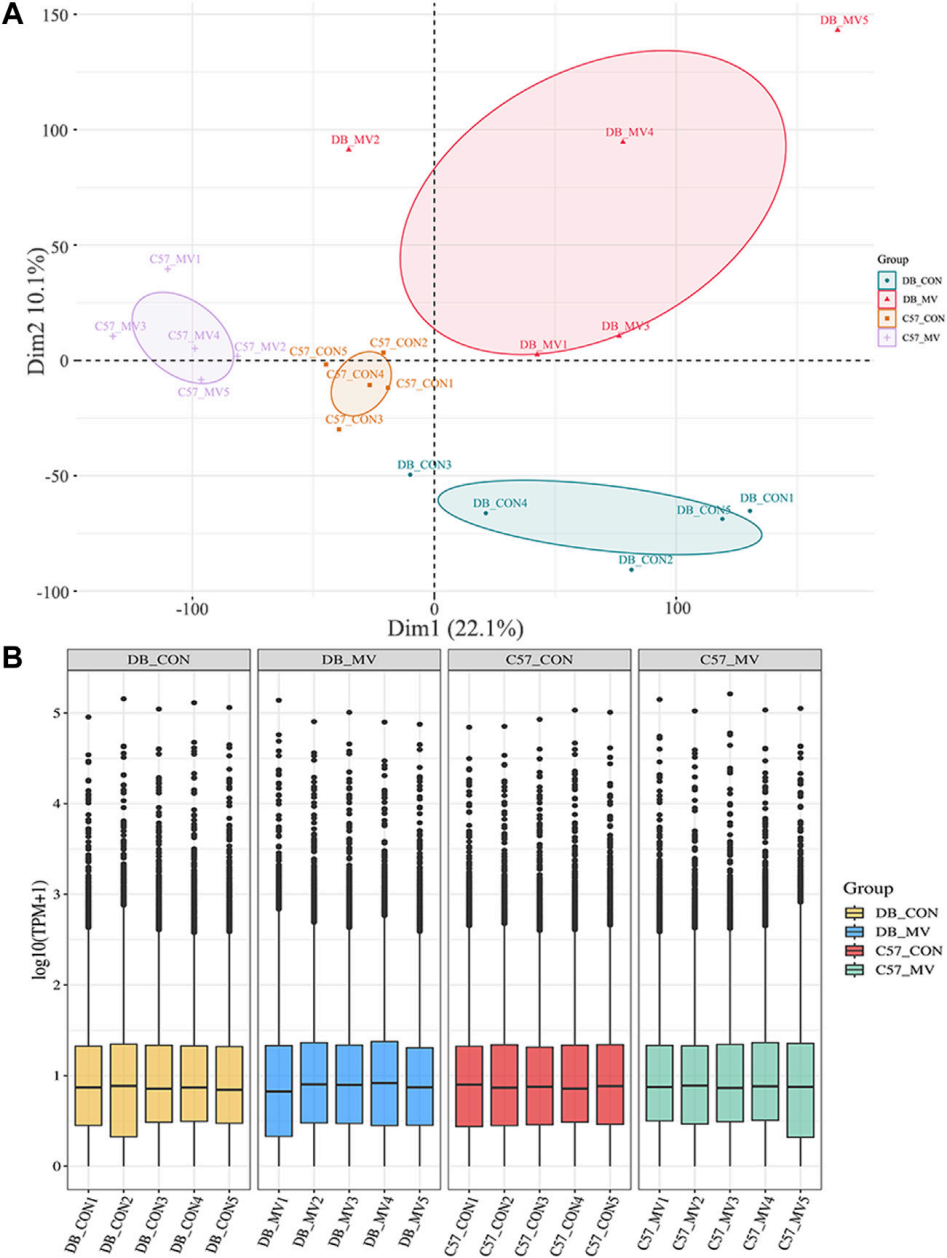
Principal component analysis and boxplot of mRNA expression in different groups. **(A)** Principal Component Analysis (PCA) between different groups. **(B)** mRNA expression levels in each group.

RNA-seq was used to identify mRNA associated with MV pathophysiological mechanisms and compare the differences in transcription levels between the MV groups of DB and C57 mice and their respective controls. Count and TPM were used as gene expression level (Supplementary Table S2). Principal component analysis (PCA) was performed to test the relationship among four groups of samples (DB-CON group, DB-MV group, C57-CON group, and C57-MV group) (Figures 2A, B). From that, we found that the confidence ovals of the samples in the control group and the MV group were separated from each other. And the result also suggests that the gene expression pattern in the same group was similar, and the difference among different groups was significant. These results show that the MV model has repeatability for VIDD, the reliability of the experiment is acceptable, and it can be further analyzed.

### 3.4 Analysis of differentially expressed genes and enrichment analysis

**FIGURE 3:**
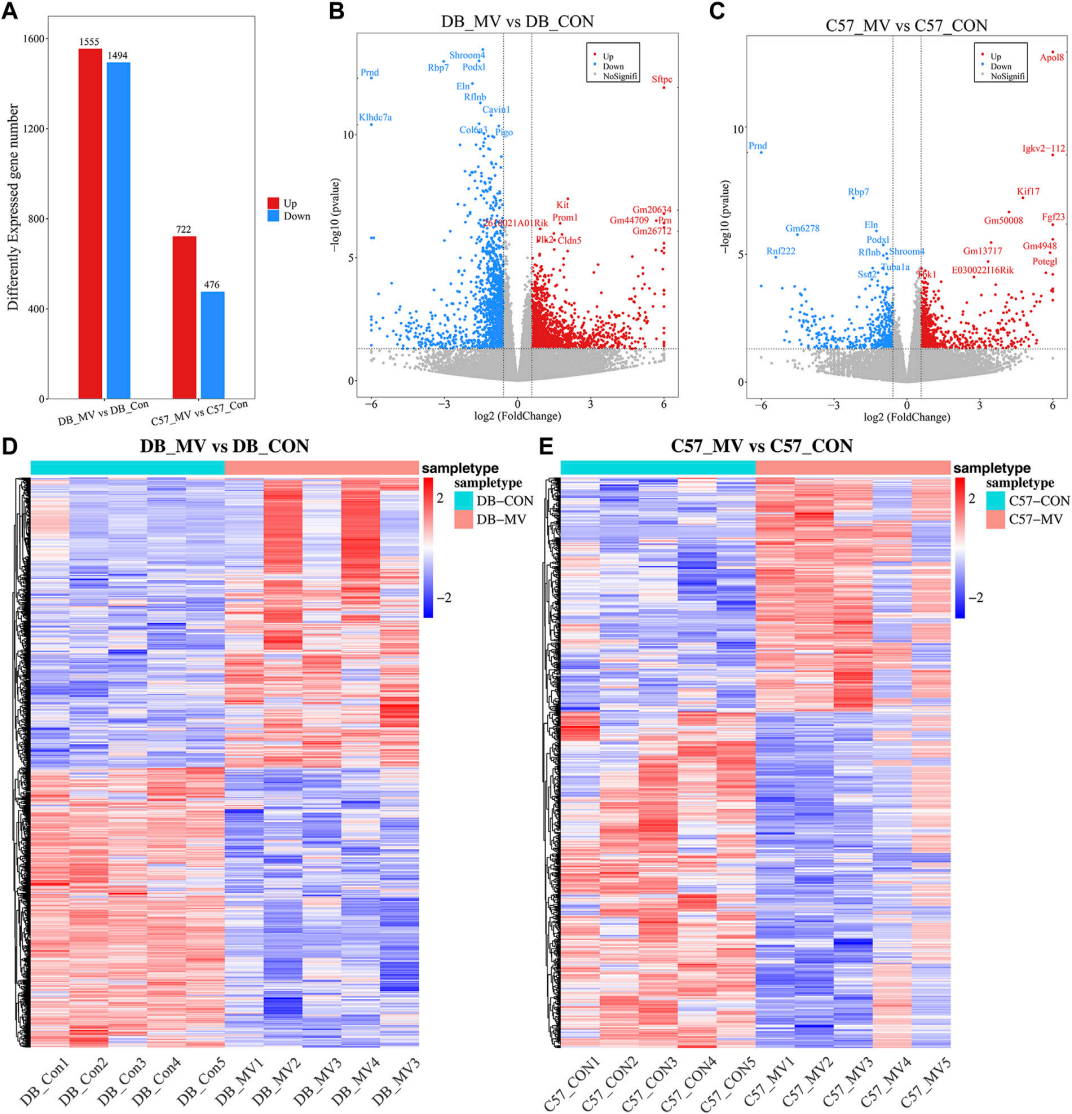
Differential gene analysis in each group of mice. **(A)** The number of DEGs in DB and C57 mice. **(B)** Volcano map of DEGs in DB mice; **(C)** Volcano map of DEGs in C57 mice; **(D)** Heat map of DEGs in DB group; **(E)** Heat map of DEGs in C57 group.

**FIGURE 4:**
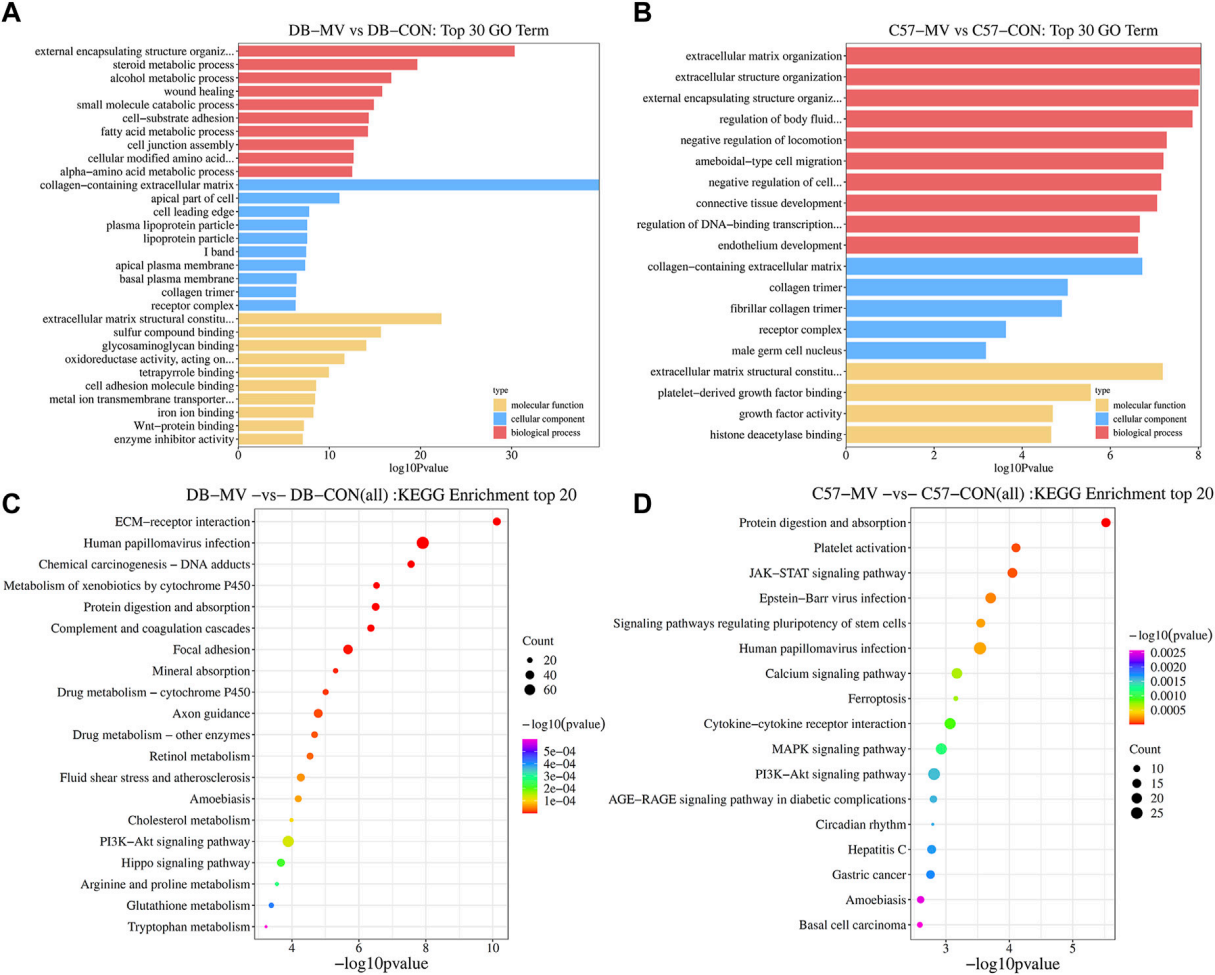
GO and KEGG analysis of DEGs in DB and C57 mice. **(A)** The top 30 GO items in DB mice group; **(B)** The top 30 GO items in C57 mice group. **(C)** The top 20 items of KEGG pathway in DB mice. **(D)** The top 20 items of KEGG pathway in C57 mice.

**FIGURE 5:**
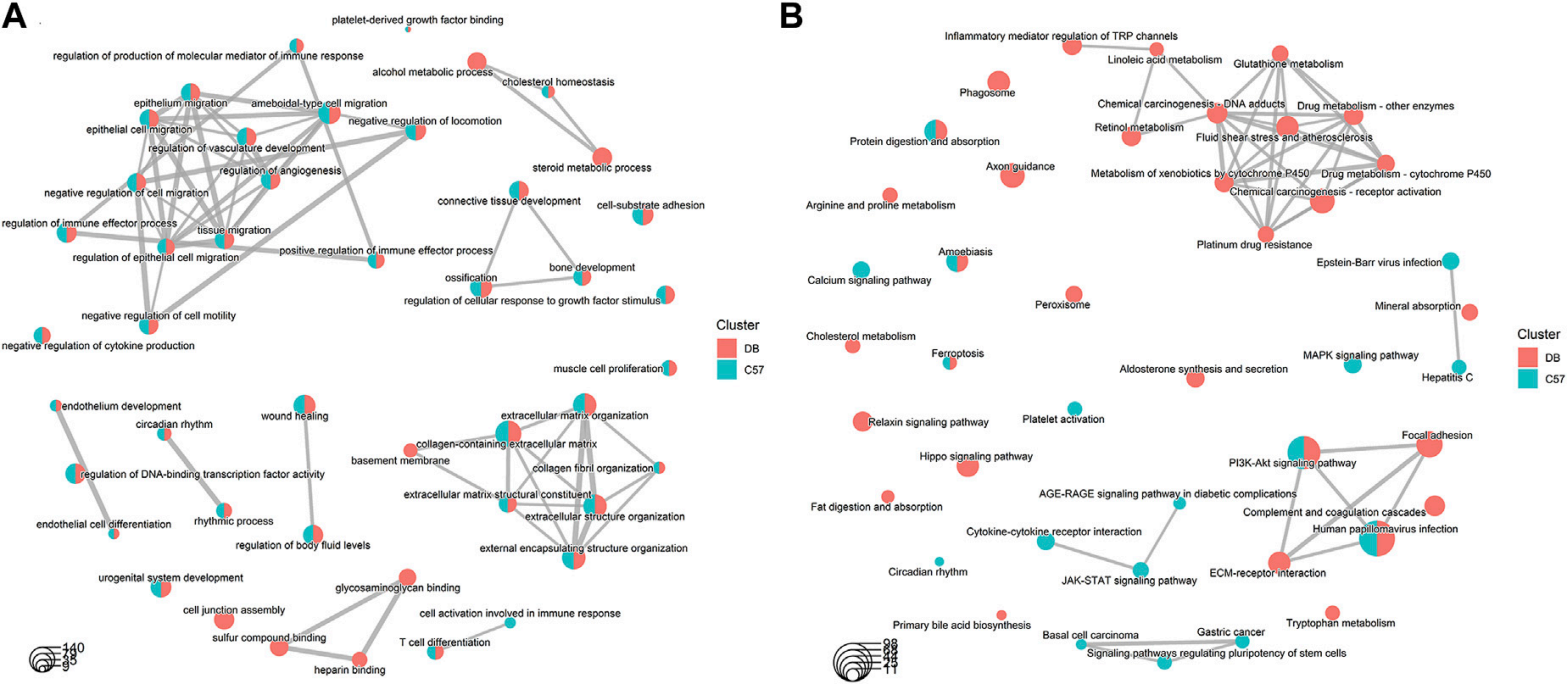
Comparative network analysis of GO and KEGG terms in DB and C57 mice. **(A)** Comparative network analysis of GO terms in DB and C57 mice; **(B)** Comparative network analysis of KEGG terms in DB and C57 mice. Red indicates the pathways included in the DB group; Green indicates the pathways included in the C57 group, and the size of the dots indicates the number of genes contained in each pathway.

The gene expression of DB and C57 mice in mechanical ventilation group and control group was analyzed. |log2FC| > 0.58 and *p*-value < 0.05 were set as threshold for differentially expressed genes (DEGs). A total of 3,049 DEGs were obtained in DB group, including 1,555 upregulated genes and 1,494 downregulated genes. And in the C57 group, a total of 1,198 DEGs were obtained including 722 upregulated genes and 476 downregulated genes (Figure 3A and Supplementary Table S3). Top 20 DEGs with the most significant *p*-value were shown in volcano maps (Figures 3B, C). The gene expression pattern of all DEGs were shown by heat maps in different groups (Figures 3D, E). In order to understand the function of DEGs in DB and C57 mice after mechanical ventilation compared with the respective control group, the GO and KEGG enrichment analysis of DEGs in DB and C57 mice were performed and the results showed that ECM, collagen-containing extracellular matrix, protein digestion and absorption, PI3K-Akt signaling pathway, etc., they are important signaling pathway changes in mechanical ventilation (Figures 4A–D and Supplementary Tables S4, S5). By further network analysis of GO and KEGG enriched signaling pathways, we can obtain specific signaling pathways that respond to mechanical ventilation in mice with diabetic background. Such as cell junction assembly, inflammatory mediator regulation of TRP channels, glutathione metabolism, metabolism of xenobiotics by cytochrome P450, relaxin signaling pathway and ECM-receptor interaction, etc. (Figures 5A, B).

### 3.5 The results of weighted gene co-expression network analysis (WGCNA)

**FIGURE 6:**
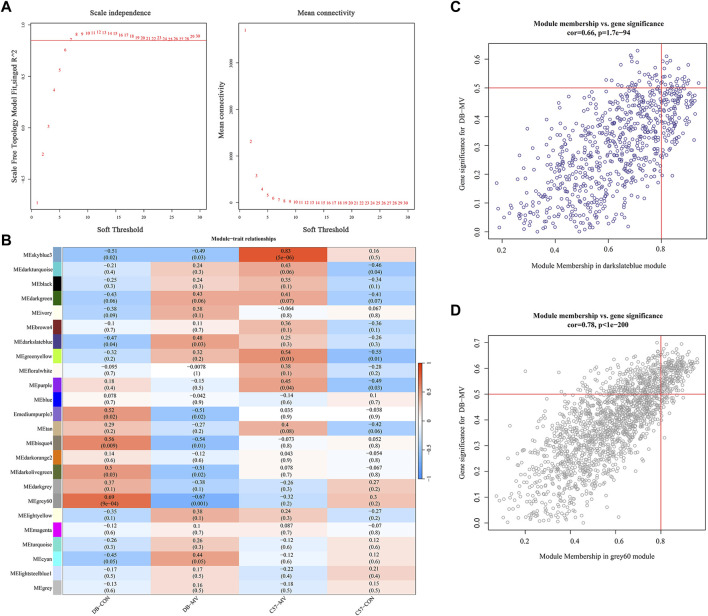
Construction and module analysis of weighted gene co-expression network analysis (WGCNA). **(A)** Network topology analysis under various soft thresholds. Left: The X-axis indicates the soft threshold. The Y-axis represents the fit index of the scale-free topological model. Right: The X-axis represents the soft threshold. Y-axis reflects average connectivity (degree). **(B)** Module-trait association. Each row corresponds to a module, and each column corresponds to a trait. Each cell contains the corresponding correlation and *p*-value. This table is color-coded according to the relevance of the color legend. **(C,D)** Associations of members in the darkslateblue module and grey60 module with trait and trait genes.

The gene expression profiles of the DB-CON, DB-MV, C57-CON and C57-MV groups were subjected to clustering analysis. To construct a co-expression network, we chose 7 as the lowest power that constructs a scale free topology (Figure 6A). Next, we converted the expression matrix to an adjacency matrix, and then converted it to a topological matrix. The average linkage hierarchical clustering method was used to cluster the genes. We also set the minimum number of genes in each gene network module to 50 according to the criteria of hybrid dynamic shear tree, which was used to identify gene modules and calculate the characteristic gene value of each module. The parameters were as follows: height (MEDissThres) = 0.25, depth division = 2, minimum module size = 50; A total of 24 modules were obtained (Figure 6B). The positive and negative correlation modules with the highest correlation with diabetes and mechanical ventilation were selected respectively (positive correlation module darkslateblue, r = 0.48, *p* = 0.03; Negative correlation module grey60, r = −0.67, *p* = 0.001). The significance of genes in darkslateblue module and grey60 module was cor = 0.66, *p* = 1.7e-94, cor = 0.78, p = 1e-200, respectively (Figures 6C, D). Genes GS > 0.5 and MM > 0.8 were selected for analysis.

### 3.6 Identification and enrichment analysis of VIDD key genes in diabetic mice

**FIGURE 7:**
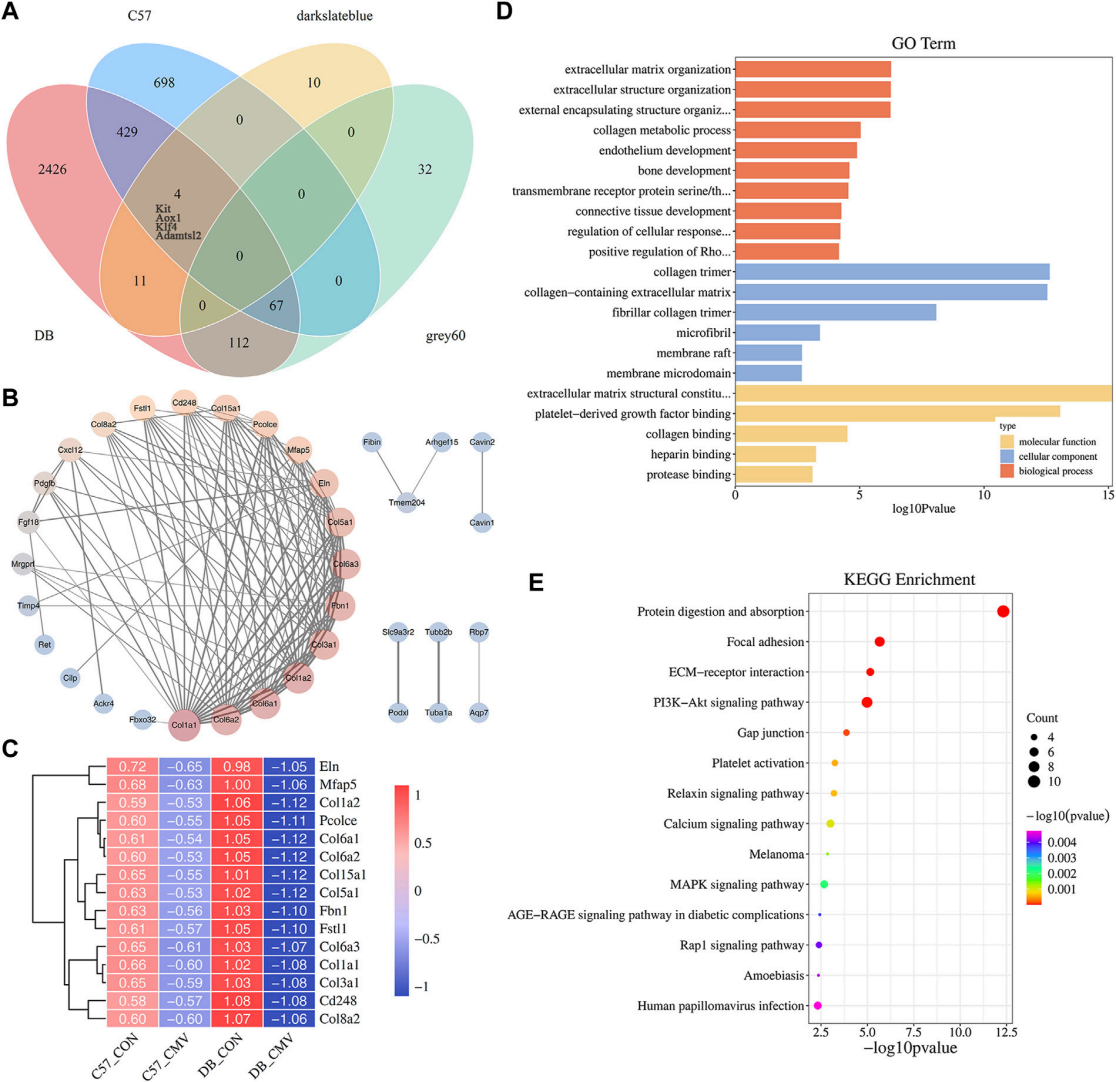
Identification and enrichment analysis of VIDD key genes in diabetic mice. **(A)** Venn diagram of crossing genes in DB and C57 mouse DEGs, WGCNA darkslateblue module, and grey60 module; **(B)** Protein interaction network analysis of the 67 genes; **(C)** The expression heat map of 15 key genes in each group of mice. **(D)** GO analysis of the 67 genes; **(E)** KEGG analysis of the 67 genes.

To identify essential genes function on the regulation of VIDD in diabetic mice, Venn analysis was performed on 3,049 DEGs in DB group, 1,198 DEGs in C57 group, 211 genes of WGCNA darkslateblue module and 25 genes of grey60 module, and finally, 67 genes were obtained (Figure 7A). Then we constructed a protein interaction network between them (Figure 7B), and the gene expression pattern of the most critical 15 genes was shown by a heat map (Figure 7C). It was found that the differentially expressed genes affecting MV in DB mice were mainly related to collagen, and the closely related collagen proteins were Col1a1, Col1a2, Col3a1, Col5a1, Col6a1, Col6a2, Col6a3, Col8a2 and Col15a1. Compared with C57 mice, the expression levels of some extracellular matrix related genes, such as collagen, elastin Eln, fibrin Fbn1, Mfap5, Pcolce, and CD248, decreased more significantly after mechanical ventilation in DB mice (Figure 7C). In addition, compared with C57 mice, the expression of Fbxo32 gene in the diaphragm of DB mice was more significantly elevated after MV (Figure 7B). GO and KEGG enrichment analysis of the 67 genes showed that the main pathways related to diabetes were extracellular matrix (ECM), protein digestion and absorption, PI3K-Akt signaling pathway, calcium signaling pathway, MAPK signaling pathway and AGE-RAGE signaling pathway in diabetic complications, etc. (Figures 7D, E and Supplementary Table S6), ECM have the closest relationship with VIDD in diabetic mice.

### 3.7 Quantitative real-time polymerase chain reaction verification

**FIGURE 8:**
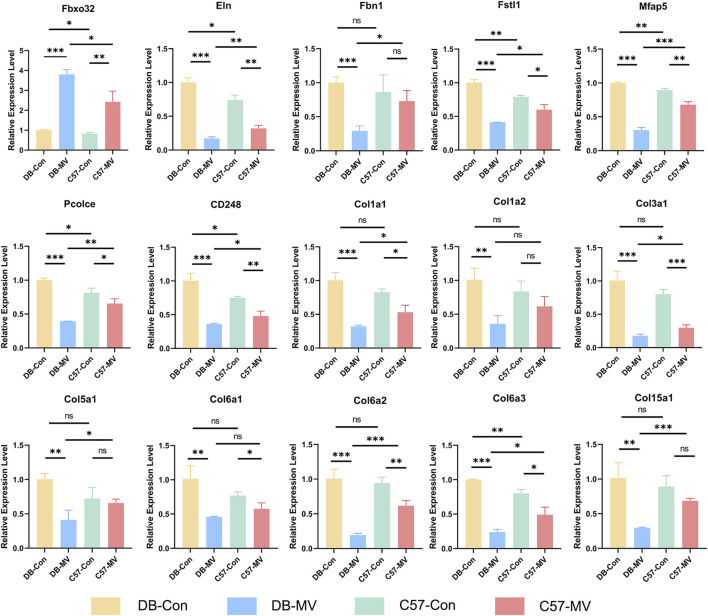
Results of qRT-PCR for selected mRNAs in four groups. The relative expression of each mRNA in each group was normalized using the DB-CON group. “*, ** and ***” are separately indicates *p*-values < “0.05, 0.01 and 0.001.” The error bars indicate the ±SD.

Next, we verified the expression of the 15 most critical genes using qRT-PCR. Comparisons between qRT-PCR results were performed using independent sample Student’s t tests. The results showed that compared with C57 mice, the expression of Fbxo32 in the diaphragm of DB mice was more significantly elevated after MV, while most of the 15 genes’ expression levels decreased more significantly after MV in DB mice (Figure 8). Although there were no significant differences in the expression of Col1a2, Col6a1, etc. between DB and C57 mice, there was a downward trend (Figure 8). In conclusion, the gene expression patterns verified by qRT-PCR were generally consistent with those detected by RNA-seq.

## 4 Discussion

In this study, we successfully established MV models in DB and C57 mice, which successfully replicated the genotypic and phenotypic characteristics of the diaphragm with impaired function and structure in diabetic mice in the MV state. Compared with mice in the C57 group, we observed significant decrease in diaphragm muscle strength and muscle fiber cross-sectional area after mechanical ventilation in diabetic mice, leading to more severe VIDD. Based on these phenotypic characteristics, we further explored DEGs in MV models of DB and C57 mice, identified key genes associated with diaphragm dysfunction in diabetic mice by WGCNA analysis, and predicted the biological function of DEGs and the signaling pathway which they participate in by GO and KEGG analysis. This may help elucidate the role of genes in the pathophysiology of VIDD in diabetic mice and provide some basis for the study and treatment of VIDD in diabetic patients.

Our study found that the muscle strength of the diaphragm and the cross-sectional area of both fast and slow-twitch fibers decreased after MV in C57 mice (Figures 1A–C), and this was more pronounced in DB mice (Figures 1A–C). Past studies showed that fast-twitch fibers are required for instantaneous exercise and have a high demand for glycolysis, while slow-twitch fibers are necessary for extended periods of exercise and utilize mitochondrial respiration and thus contain abundant mitochondria (Yasuda et al., 2023). Thus, the effect of diabetes on VIDD may be different for different muscle fibers. Wang, Y. etc. showed that disuse-related skeletal muscle atrophy that typically occurs during denervation, immobilization is primarily type I (slow-twitch) fiber-related, whereas nutrient-related atrophy such as cancer/aging cachexia, sepsis, and diabetes are more directed to type II (fast-twitch) fiber wasting (Wang and Pessin, 2013). In our study both types of muscle fibers, fast and slow, were found to be affected in the diaphragm after mechanical ventilation, and there is no significant difference between them. It is probably because oxidative stress impairs both fast and slow muscles. Liu and Li (2018) showed that MV-induced oxidative stress in the diaphragm can impair diaphragm contractility. Another study showed that elevated levels of Reactive Oxygen Species (ROS) in the diaphragm during mechanical ventilation, which in turn causes oxidative stress injury, is a prerequisite for diaphragmatic dysfunction (Zhu and Sassoon, 2012). The diaphragm is a type of striated muscle with specific physiological characteristics that make it more sensitive to events causing oxidative stress compared to other skeletal muscles (Fogarty et al., 2018). Diabetic mice showing more severe diaphragm dysfunction may be due to the higher levels of oxidative stress caused by elevated blood glucose in the mice. Studies have shown that in a model of insulin-dependent diabetes induced by streptozotocin injection (430 vs. 110 mg/dL in the hyperglycemic model and control group), compared with the control group, diaphragmatic muscle mass decreased by one-third and muscle strength decreased by 40% (Callahan LA and Supinski, 2014; Hafner et al., 2014), which was basically consistent with our findings. Previous studies have found that MV increases oxidative stress levels in the diaphragms of wild-type mice (Picard et al., 2012b). In contrast, our study found that the muscle strength and muscle fiber cross-section area of diabetic mice decreased more significantly after MV, which may be related to the hyperglycemia and oxidative stress environment of diabetic mice. At the cellular level, studies have shown that hyperglycemia can significantly enhance the level of oxidative stress, and oxidative stress can cause the release of mitochondrial ROS, resulting in mitochondrial dysfunction and aggravating diaphragm dysfunction (Leverve, 2003; Callahan LA and Supinski, 2014), indicating that hyperglycemia may lead to the deterioration of VIDD oxidative stress level, and controlling blood glucose level will help improve VIDD in diabetic mice.

To further investigate the transcriptional changes of DB and C57 mice, RNA sequencing was performed on diaphragm samples of each group. Analysis of DEGs combined with WGCNA showed that Fbxo32 (Atrogin-1) expression in the diaphragm increased after mechanical ventilation in C57 mice, and Fbxo32 in the diaphragm of DB mice increased even more (Figure 7B). Studies have shown that Fbxo32, an important transcription factor, is an important marker reflecting muscular atrophy (Liu et al., 2021b). Our previous studies on diaphragmatic in the rat VIDD model also found that the ex-pression of Fbxo32 is upregulated after MV (Liu et al., 2021b), which is consistent with the results of our current study. Fbxo32 increased more significantly in the diaphragm tissue of DB mice after MV, which provided further evidence that MV induced more severe diaphragm dysfunction and atrophy in mice with diabetes versus healthy mice.

In addition, our RNA-seq results found that the differentially expressed genes affecting MV in DB mice were mainly related to collagen, and the closely related collagen were as follows: Col1a1, Col1a2, Col3a1, Col5a1, Col6a1, Col6a2, Col6a3, Col8a2 and Col15a1, and other extracellular matrix related genes such as: elastin Eln, fibrin Fbn1, Mfap5, Pcolce, CD248 and other genes (Figures 7B, C), which decreased more significantly after MV than that in C57 mice (Figures 7B, C), suggesting that these genes may be the key genes affecting VIDD in diabetic mice. We also verified the reliability of their expression by qPCR and found that the mRNA expression levels of most genes in each group were consistent with the RNA-seq sequencing results. The reason for the non-significant difference in expression changes of Col1a2, Col6a1, etc. may be due to the small sample size of mice or the limitation of individual differences, but the trend of the gene expression levels in each group is generally consistent with the RNA-seq sequencing results, indicating that the results are basically reliable.

Studies have found that collagen is an important part of the extracellular matrix, a key protein for muscle contraction and muscle strength maintenance, and participates in the remodeling of muscle fibers and the maintenance of muscle tone (MAA, 2019). In our study we found that the collagen Col6 family accounted for the highest proportion of the major differential genes, and we predicted that they might play an important role in the diaphragm dysfunction during mechanical ventilation in DB mice. Previous studies have found that type 6 collagen has three genes encoding three main chains: Col6a1, Col6a2, and Col6a3 (Weil et al., 1988; Heiskanen et al., 1995). It is widely expressed in muscles, tendons and junctions of tendons (Hessle and Engvall, 1984; Keene and Glanville, 1988). Col6 plays a key role in maintaining skeletal muscle cell and tissue homeostasis, mediating muscle fiber anchoring from basal layer to interstitial ECM (Kuo et al., 1997; Lamandé and Bateman, 2018). At the same time, Col6 represents a finely tunable binding platform in ECM, linking several collagens (including types 1, 2, 4, 5, and 14), various glycoproteins, glycosaminoglycans, hyaluronic acid, and heparin, as well as other ECM factors and molecules (Cescon et al., 2015; Castagnaro et al., 2021). In addition, Col6 plays a variety of functions in the tissues in which it is expressed, ranging from mechanical roles (a typical collagen component in ECM) to more specific cellular protective functions (including inhibition of apoptosis and oxidative damage), regulation of cell differentiation and autophagy mechanisms (Cescon et al., 2015). Type 6 collagen, as an ECM protein, is essential for maintaining muscle and skin integrity and function. When the genes encoding their main chains, Col6a1, Col6a2 and Col6a3 are mutated, they cause congenital muscle disorders. The main manifestations of type VI collagen-related myopathy are hypotonia, muscle weakness, proximal contracture and distal relaxation (Allamand et al., 2010; Di Martino A et al., 2023; Starace et al., 2023). In our study, we found that Col6a1, Col6a2 and Col6a3 are the main differentially expressed genes in the diaphragm of diabetic mice after mechanical ventilation, and they were closely related to each other. The decreased expression may be related to the aggravation of VIDD in diabetic mice.

To further clarify the function of Col6, in the Col6a1^−/−^ mouse model, the absence of the α1 backbone leads to the complete absence of Col6 secretion in all tissues (Bonaldo et al., 1998).Studies in mice with Col6a1 deletion have shown that Col6 has a protective effect on cells because the deletion of the gene triggers spontaneous apoptosis of muscle fibrocytes, which is associated with potential mitochondrial dysfunction and organelle changes (Irwin et al., 2003). In Col6 knockout muscles, increased mitochondrial intima permeability transition pore (PTP) openings are thought to contribute to the muscular dystrophy phenotype (Angelin et al., 2007; Bernardi and Bonaldo, 2008). In addition, other studies have shown that the mitochondrial changes in Col6a1^−/−^ mouse muscles are caused by the regulatory defects of the autophagy pathway, and reactivation of the autophagy pathway through drug, genetic or nutritional pathways can rescue the dysfunction of organelles, inhibit apoptosis and prevent the decline of muscle strength (Grumati et al., 2010; Chrisam et al., 2015; Metti et al., 2021). Since the absence of Col6 can lead to muscle dysfunction or muscular atrophy (Di Martino A et al., 2023), it suggests that Col6 may be an important target for improving ventilator diaphragm dysfunction in diabetic mice.

We further performed GO and KEGG analyses to predict the function of DEGs. GO and KEGG enrichment analysis identified signaling pathways associated with VIDD in diabetic mice such as: signaling pathway, calcium signaling pathway, MAPK signaling pathway and AGE-RAGE signaling pathway in diabetic complications, etc (Figures 7D, E). In the analysis, we noticed that ECM was enriched, and it may be very closely related to VIDD in diabetic mice. Literature review found that extracellular matrix (ECM) is an important component of skeletal muscle. ECM accounts for 10% of skeletal muscle weight (Kjaer, 2004), and is located in the muscle at three levels: endomyuscular, perimuscular and epimuscular (de Rezende Pinto et al., 2015), in which a lot of collagen is distributed (Kovanen, 2012). As a special skeletal muscle, diaphragm has most of the characteristics of skeletal muscle (Zuo et al., 2000). In this study, we also found that there were different expressions of collagen in the diaphragms of different groups (Figures 7B, C). Studies have shown that ECM provides a frame structure for muscles, supporting muscle fibers, capillaries, and nerves. It plays an important role in the transmission, maintenance and repair of muscle fiber force (Gillies and Lieber, 2011; MAA, 2019). ECM deficiency can lead to muscular dystrophy or muscular atrophy (Schessl et al., 2006). In this study, the main components of ECM in DB mice decreased more significantly than C57 mice after mechanical ventilation, such as collagen, elastin and fibrillar protein, indicating that some components of ECM were insufficient or degraded too much during mechanical ventilation, resulting in diaphragmatic atrophy, which was manifested as more serious diaphragmatic dysfunction in DB mice.

## 5 Conclusion

In conclusion, our study provides a groundbreaking glimpse into transcriptional level changes in diaphragm function after MV in DB mice and C57 mice. Based on our results, ECM is an important focus of ventilator-associated diaphragm dysfunction in diabetic mice, providing clues for the further study of mechanical ventilation in diabetic mice. Col6 may be a promising target for diagnosis and treatment of ventilator diaphragmatic dysfunction in diabetic mice. Future research could delve into these aspects, driving a more complete understanding of the impact of diabetes on MV and ultimately guiding treatment innovation.

## Data Availability

The datasets presented in this study can be found in online repositories. The names of the repository/repositories and accession number(s) can be found below: https://www.ncbi.nlm.nih.gov/sra/PRJNA1055679.
