# Supplementary material for: Identification of key genes affecting ventilator-induced diaphragmatic dysfunction in diabetic mice
Source: Front Genet. 2024 May 9;15:1387688. doi: 10.3389/fgene.2024.1387688 (PMC11112022; doi:10.3389/fgene.2024.1387688)
Supplement: Supplementary file 3 [file Table1.DOCX]

Table S1 Primers designed for qRT-PCR validation of mRNAs.

| **mRNA** | **Primer type** | **Primer Sequence (5’-3’)** |
| --- | --- | --- |
| Eln | Forward | TTGCTGATCCTCTTGCTCAAC |
| Eln | Reverse | GCCCCTGGATAATAGACTCCAC |
| Mfap5 | Forward | GTCTTGGCAATCAGCATCCC |
| Mfap5 | Reverse | CCAGATTAGGGTCGTCTGTGAAT |
| Col1a2 | Forward | AGTCGATGGCTGCTCCAAAA |
| Col1a2 | Reverse | CCTCAGTTCGTGTCAGCCTT |
| Pcolce | Forward | GCCAGACCCCCAACTACAC |
| Pcolce | Reverse | CCGTAATTGTCCAGATGCACTT |
| Col6a1 | Forward | AACAGGAATAGGAAATGTGACCC |
| Col6a1 | Reverse | ACACCACGGATAGGTTAGGGG |
| Col6a2 | Forward | AAGGCCCCATTGGATTCCC |
| Col6a2 | Reverse | CTCCCTTCCGACCATCCGAT |
| Col15a1 | Forward | CTCTGGCCAGCAAGGAATGA |
| Col15a1 | Reverse | GGTGGGTCCTGAGATTGTGG |
| Col5a1 | Forward | CTTCGCCGCTACTCCTGTTC |
| Col5a1 | Reverse | CCCTGAGGGCAAATTGTGAAAA |
| Fbn1 | Forward | GGACGCCAATTTGGAGGCT |
| Fbn1 | Reverse | CTTTCAGCGCATCGTGTCCT |
| Fstl1 | Forward | CACGGCGAGGAGGAACCTA |
| Fstl1 | Reverse | TCTTGCCATTACTGCCACACA |
| Col6a3 | Forward | GCTGCGGAATCACTTTGTGC |
| Col6a3 | Reverse | CACCTTGACACCTTTCTGGGT |
| Col1a1 | Forward | GCTCCTCTTAGGGGCCACT |
| Col1a1 | Reverse | CCACGTCTCACCATTGGGG |
| Col3a1 | Forward | ACGTAGATGAATTGGGATGCAG |
| Col3a1 | Reverse | GGGTTGGGGCAGTCTAGTG |
| Cd248 | Forward | CAACGGGCTGCTATGGATTG |
| Cd248 | Reverse | GCAGAGGTAGCCATCGACAG |
| Col8a2 | Forward | GAGCGACGCGGAGTTCTG |
| Col8a2 | Reverse | GCGTCTGTGGATGGGCTTTT |
| Fbxo32 | Forward | CAGCTTCGTGAGCGACCTC |
| Fbxo32 | Reverse | GGCAGTCGAGAAGTCCAGTC |
| GAPDH | Forward | AGGTCGGTGTGAACGGATTTG |
| GAPDH | Reverse | TGTAGACCATGTAGTTGAGGTCA |
